# Supplementary material for: A Sec14-like phosphatidylinositol transfer protein paralog defines a novel class of heme-binding proteins
Source: eLife. 2020 Aug 11;9:e57081. doi: 10.7554/eLife.57081 (PMC7462610; doi:10.7554/eLife.57081)
Supplement: Figure 3—source data 1. — The S. cerevisiae Sfh5 primary sequence was used as query in a Uniprot BLAST search to identify orthologs across the fungal kingdom. Initial sequence alignments were performed using ClustalW and followed by a Sfh5 structure guided alignment of the sequences using ESCRIPT. Sequences from C. glabrata, C. albicans, C. tropicalis, C. auris, A. nidulans, A. oryzae, N. crassa, C. neoformans and Y. lipolytica were aligned. Conserved residues are highlighted in red. At bottom are identified the Sfh5 residues that: (i) correspond to the PtdIns-binding barcode common to Sec14-like PITPs (▲), (ii) that are engaged in van der Waals interactions with heme or π-π stacking interactions with the pyrrole groups of the porphyrin ring (●), (iii) engage in electrostatic or H-bond interactions with heme -- that is. the heme-binding barcode (♦) and (iv) conserved residues Lys188 and Lys192 that are poised to gate access to the empty pocket distal to the heme iron (arrows). Secondary structure elements as determined from the Sfh5 crystal structure are identified at top. [file elife-57081-fig3-data1.pdf]

Figure 3 -- figure supplement 1

```
S_cerevisiae_Sfh5

S_cerevisiae_Sfh5              ............................................................
C_glabrata_Sfh5                ............................................................
C_albicans_Sfh5                ............................................................
C_tropicalis_CTRG_03339        ............................................................
C_auris_B9J08_004048           ............................................................
A_nidulans_An8233              MAEQAKLPDQAQPVPETQVPDNGKPEQQPTATESAPAPEPATTEPTTAATAPSAVDGTGE
A_oryzae_Ao090102000579        .MADQQEKTASSAVPETQPPS..QTAETTTQTTATPAPEVQTEQTQPTESGPSAAN.TTT
N_crassa_NCU02200              ...........................MSTQPSDSAEPGAAPAAVSTYAGVENTPTVPGS
C_neoformans_CNE04320          ............................................................
Y_lipolytica_YALI0_D08866g     ............................................................

                                                                            α1
S_cerevisiae_Sfh5                                                  1        10        20

S_cerevisiae_Sfh5              ..................................MKFDNDSEKQVFDKLKKAIPGIIKEK
C_glabrata_Sfh5                ..................................MKFENDNEKQVFDKLSRSLGNIIETK
C_albicans_Sfh5                ....................MSSSIEEVKATIKSVKLTDSQAEKLSKLIDSLPKILSGLD
C_tropicalis_CTRG_03339        ....................MTTSIEEVKSTIKSTELKDDEAEKLAKLISSIPDILSKVD
C_auris_B9J08_004048           ............................MALVKSTKLTEEQEKKLQQLIQDIPDILAKTE
A_nidulans_An8233              TAPAAPEPAAAPVAAAAAAPAPEPTKSEPQPAVGEQSEPAKKDEPAKPEYFTKTPALEQF
A_oryzae_Ao090102000579        EQPTNPPAAEASKENAAPAPAPAAEDAPSEPAPAQEQQKEEKPADNKPEYLAKNPALSQL
N_crassa_NCU02200              EPDAAKHAEEEPKVVNPTEPQPTAPVDNEPKPAAAPAQEADSPADIKDSVSTTAGELSPL
C_neoformans_CNE04320          .....MSAVEAPSASQAIWP.............................ELTEDHPLSQL
Y_lipolytica_YALI0_D08866g     ..............................MSIPAHLQILQPSELEALKKLQDELPKILE

                                   β Turn β          α2         α3                  α4
S_cerevisiae_Sfh5              30        40        50        60        70        80

S_cerevisiae_Sfh5              CAGYDELYGYKLNPEGLTQEEVDKYYDEKIADRLTYKLCKAYQFEYSTIVQNLIDILNWR
C_glabrata_Sfh5                CDGYDELYGHKICKEIGSADEVQQYYNEDIAQSLLFKLCKAYQFNYDEIVTHLVKILKWR
C_albicans_Sfh5                NPEYDEIFGYRINTK......DKPYVDESIRNEILLKFLAADDYNLELSEKRLIDSLNWR
C_tropicalis_CTRG_03339        NPEYDEIFGHRINTS......DKQYVNEAIRNEILLKFLAADAYDLDLAIKRIIQTLNWR
C_auris_B9J08_004048           DPSYDEIYGYRIAPE......EKEYVNVEIRNEILLKFLIAREYDIEKAKEMLVNTFNWR
A_nidulans_An8233              FDRLPTILSNTGHQEMWGVPLKHEVTDIP.TINVLIKFLRANAGDLKAAEDQLSKALTWR
A_oryzae_Ao090102000579        FDRLPTVLSNSGHDEMWGVPLR.DSSDVP.TVNVLIKFLRANEGNVKLAEDQLTKALQWR
N_crassa_NCU02200              AQLWKAAEG.HAHFEIWGVPLSDPERHIP.TQIIFQKFLNANEGQVEKAKDQLLKTLDWR
C_neoformans_CNE04320          NSRLPTILSEAGHSQIWGVTLTYSTPPTFSSLIILQKFLRSVDNNVDEAATALGKTLKWR
Y_lipolytica_YALI0_D08866g     KTDYNEMYGHKLSEAEDG...PGKAYSEVKRDIILLKFLKARDYDIAQTKDMLTDALKWR

                                    T1          T2    β1     TT    TT           β2
S_cerevisiae_Sfh5               90        100       110       120                 130

S_cerevisiae_Sfh5              REFNPLSCAYKEVHNTELQNVGILTFDANGDANKK..........AVTWNLYGQLVKKKE
C_glabrata_Sfh5                KKFNPLSCAFKETHNKELEDVGILTWYPEEEPNKR..........VVTWNLYGKLVKKKE
C_albicans_Sfh5                NEFQPLSAAFEETFDKELNELGVITNFP..NSNLK..........ITTWNLYGNLKNPKK
C_tropicalis_CTRG_03339        NEFQPLSAAFDETFDDELNELGVVTDFS..KSKLH..........VTTWNLYGNLKNPKK
C_auris_B9J08_004048           RKFLVLSAAYDEKFDKDLEKMGVITNYEGNKDNFS..........VVTWNLYANLKNPKK
A_nidulans_An8233              KENDPIALADASKNSYDASKFKGLGYLTTYQREGK.......GDLVVTWNIYGAVKKFDE
A_oryzae_Ao090102000579        KQTRPTALVEG...RYSAKKFGGLGYLSTYKDADG.......KETVITWNIYGGVKDLGT
N_crassa_NCU02200              QKTQPQQLLRK...MFSKAKFDGLGYVTTYTAGDEPAVDEPEQKEVFTWNLYGSVKSLDE
C_neoformans_CNE04320          KDWGLDARADKKEKENFGPDFEGLGYVTKIKKNDG.......GDEIVTWNVYGAVKDLKS
Y_lipolytica_YALI0_D08866g     KEFDPLDCASAKHDSKFDKLGVITDKGAGGEPQVTN............WNLYGAVSNRKE

                                T3                  α5              TT                β3
S_cerevisiae_Sfh5                140                 150       160                 170

S_cerevisiae_Sfh5              LFQNVDK..............FVRYRIGLMEKGLSLLDFTSSDNNY...........MTQ
C_glabrata_Sfh5                LFKDVQK..............FLRYRIGLMEKGIQLLNFQDEENCY...........MTQ
C_albicans_Sfh5                IFEKFGANNKV...SKLPGSQFLRWRVGLMEKSLQLIDFTSTTDNR...........IAQ
C_tropicalis_CTRG_03339        IFEKFGANNKN...VELPGSQFLRWRIGLMEKSLQLIDFTNKDNNR...........IAQ
C_auris_B9J08_004048           LFAKFGVGVDNPDKTDLPGTMFLRWRVGLMERSLALLDFTNPDKCK...........IAQ
A_nidulans_An8233              TFGDITE..............FIKWRAALMELAVQELKLDQATSVI..DYDGEDPYQMIQ
A_oryzae_Ao090102000579        TFGNVDE..............FINWRVALMELAVKDLKMDQATSVI..DYEGEDPYQMIQ
N_crassa_NCU02200              TFGNLQE..............FVEWRVALMELGLMEINIGGAIKPI..TAD.YDPYKMTQ
C_neoformans_CNE04320          TFGDLDR..............FLRWRVNLMEEAIAHLHLATTSTPIPDFNAGIDPHRMAQ
Y_lipolytica_YALI0_D08866g     IFGDLKG..............FLRWRVGIMERSLALLDFTKPGAGS..........MLLQ
```

Figure 3 -- figure supplement 1 (….continued)

```
                                          α6                 β4         α7
S_cerevisiae_Sfh5           →  TT  TT                        →
                                 180       190       200       210       220
S_cerevisiae_Sfh5           VHDYKGVSVWRMDSDIKNCSKTVIGIFQKYYPELLYAKYFVNVPTVFGWVYDLIK..KFV
C_glabrata_Sfh5             VHDYKTVSVWRMDSDMKSCVKEVINTFQTYYPELLYAKYFVNVPSVFAWAYDIIK..TFV
C_albicans_Sfh5             VHDYNNVSMFKIDPGMKKATKEIITIFGANYPELLSTKFFINVPLIMGWVFTFFKTIRVI
C_tropicalis_CTRG_03339     VHDYNNVSILRIDPGMKKATKEIITIFGDNYPELLSTKFFINVPLLMGWVFTFFKTIGVI
C_auris_B9J08_004048        VHDYNNVSMFRMDPGMKAATKEIITIFGDNYPELLSKKYFINVPLLMGWVFAFFKATGFM
A_nidulans_An8233           VHDYLNVSFLRMNPNVKAATKKTIDVFSTAYPELLREKFFVNVPAIMGWMFAVMK..VFV
A_oryzae_Ao090102000579     VHDYLNVSFLRMNPSVKAATKKTIDVFATAYPELLREKFFVNVPSIMGWMFAAIK..VFL
N_crassa_NCU02200           VHDYKGISFLRQTDVAKAASKECIKVLGDNYPELLKEKFFLNIPAIMGFFYGLMK..MFV
C_neoformans_CNE04320       VHLYEGVSFLRMDPHVKAASKATIELMAANYPELLSRKFFVGVPLIMSWMFQAVR..MFV
Y_lipolytica_YALI0_D08866g  IHDYKNVSFLRLDAETKAASKETIRVFQSYYPETLERKFFVNVPTLMQFVFGFVN..KFL

                             T4    β5      T5
S_cerevisiae_Sfh5                  →TT                                         TTT
                           230       240       250                 260         270
S_cerevisiae_Sfh5           DETTRKKFVVLTDGSKLGQYLK........DCPYEGYGG.....KDKKNNLTKQNVTNVH
C_glabrata_Sfh5             DENTRKKFVVLNDGKKLGKYLK........QCPGDQFGG.....SSK.STIFEQNVKKVK
C_albicans_Sfh5             TEATLKKFQVLNHGNLSESFNP........DELPKVYGG.....KVEKSLFDIDVSDDIK
C_tropicalis_CTRG_03339     SQETLNKFQVLNHGDLSEFFSK........SELPKSYGG.....EVDQTLFKLDIETQIK
C_auris_B9J08_004048        SAATLKKFEMLNHGDLSEAFGK........ENLPQAYNGGETNEKFPDIFASQVDDSTIE
A_nidulans_An8233           NQNTARKFHPISNGANLAKEFP....AGVAEKFPKAYGGSAPDLESSARTVALKEVKE..
A_oryzae_Ao090102000579     SKNTTRKFHPISNGANLAREFP....PAVKEQFPKVYGGSAPDLHEGARTVALEEDNEPA
N_crassa_NCU02200           SKKTLNKFHPMSSGTNLAKEFVNTKVDGLGDKLPAEYGGKGADLKTLGKAPIVT......
C_neoformans_CNE04320       SAETAKKFVVISYKENLANELG......ELEGVPKEYGGKGLSLGELQNQLRGEDAVTSS
Y_lipolytica_YALI0_D08866g  SRETVAKFVVYSNGKDLHKSLG........SWVPAEYGGKGGPLLEMAISDHPVQEAETK

                                  α8
S_cerevisiae_Sfh5
                             280           290
S_cerevisiae_Sfh5           PTEYGGLYILQKQIIEDVE.........................................
C_glabrata_Sfh5             PTAYASYLLERQEIEDVE..........................................
C_albicans_Sfh5             LSEYGEVILKKVGDEEINHINDDVE...................................
C_tropicalis_CTRG_03339     LSEYGEVILKKVGDEEINNINNEVE...................................
C_auris_B9J08_004048        SPEYGSIILEKLKKAQEEESKKQELAEPVEKTTEGANIATDSKITEDAGPETPDDEKVTK
A_nidulans_An8233           ...EKKEEPKEGSKEEQKGEQKGE....................................
A_oryzae_Ao090102000579     PAPAAPAEPTEEAKPEQEAPKQEPAPEAPKEEAIKEALVEAPKEEPKQPAVEEPAKTDTA
N_crassa_NCU02200           ............................................................
C_neoformans_CNE04320       ............................................................
Y_lipolytica_YALI0_D08866g  ETKEKEKETEKPVGTSETDTVGDAAEPAPTEPAAETVPTTEPAPTEPAAETAPTESSTAP

S_cerevisiae_Sfh5

S_cerevisiae_Sfh5           ....................
C_glabrata_Sfh5             ....................
C_albicans_Sfh5             ....................
C_tropicalis_CTRG_03339     ....................
C_auris_B9J08_004048        KDETVA..............
A_nidulans_An8233           ....................
A_oryzae_Ao090102000579     VTTQETVAPAEAK.......
N_crassa_NCU02200           ....................
C_neoformans_CNE04320       ....................
Y_lipolytica_YALI0_D08866g  SAEPAAETAEPVSEGAKVAAQ
```
