# Supplementary material for: A Sec14-like phosphatidylinositol transfer protein paralog defines a novel class of heme-binding proteins
Source: eLife. 2020 Aug 11;9:e57081. doi: 10.7554/eLife.57081 (PMC7462610; doi:10.7554/eLife.57081)
Supplement: Supplementary file 1. [file elife-57081-supp1.docx]

**SUPPLEMENTARY MATERIALS**

**A Sec14-like Phosphatidylinositol Transfer Protein Paralog Defines a Novel Class of Heme-binding Proteins**

**Danish Khan^1^, Dongju Lee^2^, Gulcin Gulten^1^, Anup Aggarwal^1^, Joshua Wofford^3,4^, Inna Krieger^1^, Ashutosh Tripathi^2^, John W. Patrick^3^, Debra M. Eckert^5^, Arthur Laganowsky^3^, James Sacchettini^1^, Paul Lindahl^1,2^, Vytas A. Bankaitis^1,2,3*^**

**^1^Department of Biochemistry & Biophysics**

**Texas A&M University**

**College Station, Texas 77843-2128, USA**

**^2^Department of Molecular and Cellular Medicine**

**Texas A&M Health Sciences Center**

**College Station, Texas 77843-1114, USA**

**^3^Department of Chemistry**

**Texas A&M University**

**College Station, Texas 77843-2128**

**^4^Department of Chemistry**

**Charleston Southern University**

**North Charleston, South Carolina 29406**

**^5^Dept of Biochemistry**

**University of Utah School of Medicine**

**Salt Lake City, UT 84112**

**SUPPLEMENTARY TABLES**

**Supplementary Table 1.**

**Soret Sfh5 Residue Heme Content**

**Protein Maximum 173,175 Pair % Total Protein Bound**

**Sfh5 404 nm H,Y 29.7*/34.7#**

**H_173_A 403 nm A,Y 12.0*/21.0#**

**H_173_Y 402 nm Y,Y 37.0***

**Y_175_A n.d. H,A 0.48***

**Y_175_F n.d. H,F n.d*/0.01#**

**Y_175_H 407 nm H,H 11.6***

**Y_175_F,Y_68_A 413 nm H,F 2.0***

Soret peak maxima and heme content of wild-type and various mutant proteins as determined by pyridine hemochromagen assay (*) or ICP-MS (#). Not detectable (n.d.).

**Supplementary Table 2.**

**Protein Activity (units/mg)**

**Horseradish Peroxidase 1150.0**

**Sfh5 5.2**

**Sfh5^Y175H^ 20.2**

**Sfh5^H173Y^ 1.9**

Peroxidase activity measurements for Sfh5 and mutant proteins. A unit of peroxidase activity is defined as that amount of activity that produces 1 mg of purpurogallin in a 20 sec incubation at pH 7.0 at room temperature. Activities were normalized to protein heme content as determined by ICP-MS and pyridine hemochromagen assays.

**Supplementary Table 3.**

**Fe^3+^LA (large A) Fe^3+^SA (small A) Fe^2+^**

**Spin 5/2 5/2 2**

**δ (mm/s) 0.40 0.37 0.57**

**ΔE_Q_ (mm/s) 0.55 0.91 1.73**

**D (cm^-1^) 2.5 2.0 ---**

**E/D 0.072 0.067 ---**

**Ax/g_n_β_n_ (kG) -57 -121 ---**

**Ay/g_n_β_n_ (kG) -171 -58 ---**

**Az/g_n_β_n_ (kG) -216 -14 ---**

**η 0.83 0.0 ---**

**Г (mm/s) 0.48 0.45 0.37**

Mössbauer parameters used in fitting the Sfh5 and Sfh5^H173A^ spectra are identified. Instruments were calibrated using an α-Fe foil at room temperature. Approximately 800 µl of recombinant proteins (concentration = 1.5 mM) were collected in a Mössbauer cup, frozen over liquid nitrogen and stored at -80°C until use.
